# Can inertial measurement unit sensors evaluate foot kinematics in drop foot patients using functional electrical stimulation?

**DOI:** 10.3389/fnhum.2023.1225086

**Published:** 2023-11-09

**Authors:** Francesca d'Andrea, Paul Taylor, Kai Yang, Ben Heller

**Affiliations:** ^1^Sports EngineeringResearch Group, Sport and Physical Activity Research Centre, Advanced Wellbeing Research Centre (AWRC), Sheffield Hallam University, Sheffield, United Kingdom; ^2^The National Clinical FES Centre, Department of Clinical Science and Engineering, Salisbury District Hospital, Salisbury, United Kingdom; ^3^Faculty of Health and Social Science, Bournemouth University, Poole, United Kingdom; ^4^Odstock Medical Limited, Salisbury District Hospital, Salisbury, United Kingdom; ^5^Etexsense, Southampton, United Kingdom; ^6^Winchester School of Art, University of Southampton, Southampton, United Kingdom

**Keywords:** drop foot, foot kinematics, functional electrical stimulation, gait analysis, inertial measurement unit

## Abstract

The accuracy of inertial measurement units (IMUs) in measuring foot motion in the sagittal plane has been previously compared to motion capture systems for healthy and impaired participants. Studies analyzing the accuracy of IMUs in measuring foot motion in the frontal plane are lacking. Drop foot patients use functional electrical stimulation (FES) to improve walking and reduce the risk of tripping and falling by improving foot dorsiflexion and inversion-eversion. Therefore, this study aims to evaluate if IMUs can estimate foot angles in the frontal and sagittal planes to help understand the effects of FES on drop foot patients in clinical settings. Two Gait Up sensors were used to estimate foot dorsi-plantar flexion and inversion-eversion angles in 13 unimpaired participants and 9 participants affected by drop foot while walking 6 m in a straight line. Unimpaired participants were asked to walk normally at three self-selected speeds and to simulate drop foot. Impaired participants walked with and without FES assistance. Foot angles estimated by the IMUs were compared with those measured from a motion capture system using curve RMSE and Bland Altman limits of agreement. Between participant groups, overall errors of 7.95° ± 3.98°, −1.12° ± 4.20°, and 1.38° ± 5.05° were obtained for the dorsi-plantar flexion range of motion, dorsi-plantar flexion at heel strike, and inversion-eversion at heel strike, respectively. The between-system comparison of their ability to detect dorsi-plantar flexion and inversion-eversion differences associated with FES use on drop foot patients provided limits of agreement too large for IMUs to be able to accurately detect the changes in foot kinematics following FES intervention. To the best of the authors' knowledge, this is the first study to evaluate IMU accuracy in the estimation of foot inversion-eversion and analyze the potential of using IMUs in clinical settings to assess gait for drop foot patients and evaluate the effects of FES. From the results, it can be concluded that IMUs do not currently represent an alternative to motion capture to evaluate foot kinematics in drop foot patients using FES.

## 1. Introduction

Drop foot is a syndrome affecting patients suffering from neurological conditions such as stroke, multiple sclerosis, Parkinson's disease, and spinal cord trauma. It is characterized by a decreased range of motion of the ankle in the three planes of motion. In particular, subjects present a limited ability to dorsiflex the ankle by voluntary muscle activation and a tendency to invert the foot during the swing phase of gait (Stevens et al., 2015). This results in reduced foot clearance, increased cadence, and ankle instability at initial contact due to poor placement of the foot on the ground (Knutsson and Richards, 1979; Brandstater et al., 1983). To reduce toe catching during swing and the consequent risk of tripping and falling, people with drop foot adopt compensatory strategies such as hip hitching, leg circumduction, and foot lifting. These movements increase clearance but also energy expenditure (Malešević et al., 2017).

Functional electrical stimulation (FES) is used as a treatment to improve walking in people with drop foot, enabling them to walk at least 5 m with or without the aid of a walking stick (Burridge et al., 1997). Common FES systems consist of a single-channel stimulator with skin surface electrodes attached over the peroneal nerve and the tibialis anterior motor point. Stimulation is triggered by a pressure-sensitive heel switch placed inside the shoe under the heel and induces a nerve impulse through the two electrodes that propagates along the muscles, producing a response similar to a normal contraction during the swing phase of walking. These contractions correct drop foot by increasing foot clearance during the swing phase of walking and ensuring that the foot contacts the ground at the end of the swing phase with the heel first and in a slightly everted position, contributing to greater ankle stability and optimal weight bearing through the center or slightly medially to the center line of the foot during the stance phase (Taylor and Street, 2015). Each individual's response to FES is different, varies with time due to muscular fatigue and time-variant spasticity, and is very sensitive to electrode positioning and channel intensities, which are crucial to achieving a well-balanced dorsiflexion without excessive inversion or eversion (Veltink et al., 2003; Seel et al., 2016; Schauer, 2017). Therefore, the evaluation of foot motion is fundamental to understanding the effect of FES and any rehabilitative benefits.

Motion capture systems are the gold standard for biomechanical assessments and are widely used to analyze foot kinematics during different movement tasks, such as walking (Sun et al., 2018), running (Li et al., 2022), and jump landings (Azevedo et al., 2020). However, motion capture systems are expensive, complex, time-consuming to set up, and sensitive to light; hence, measurements are usually restricted to specialist laboratories with trained personnel and may not be appropriate for busy clinical settings delivering FES interventions for drop foot. Inertial measurement units (IMUs) represent an alternative to motion capture systems to analyze gait in a clinical setting, primarily due to their lower costs, small size, insensitivity to the environment, and ease of use and set-up.

IMUs have been previously used and validated against gold-standard measurement systems to evaluate spatiotemporal parameters, foot clearance, and foot motion. Spatiotemporal parameters have been validated for healthy participants of different ages (Mariani et al., 2010; Brégou Bourgeois et al., 2014; Zhou et al., 2020), participants with neurological or orthopedic diseases (Mariani et al., 2013; Brégou Bourgeois et al., 2014; Sijobert et al., 2018; Lefeber et al., 2019; Laidig et al., 2021), and healthy participants with asymmetry induced by the alteration of the thickness of a shoe sole (Schwameder et al., 2015). The estimation of foot clearance has been validated and used to compare children with and without cerebral palsy (CP) (Brégou Bourgeois et al., 2014) and healthy adults of various age groups (Mariani et al., 2010; Kanzler et al., 2015). The accuracy of the estimation of dorsi-plantar flexion using IMUs has been analyzed for healthy participants of different age groups (Kanzler et al., 2015; Schwameder et al., 2015), participants with neurological conditions (Brégou Bourgeois et al., 2014; Sijobert et al., 2018), and amputees (Seel et al., 2015). Two studies (Seel et al., 2014; Falbriard et al., 2020) evaluated foot inversion-eversion, but neither of the studies evaluated the accuracy of the measurements in comparison to a gold standard system. An accurate estimation of foot motion in the sagittal and frontal plane is necessary to understand drop foot patients' responses to treatment and rehabilitation with FES.

Older adults and patients affected by neurological or orthopedic conditions, compared to younger and healthy populations, walk at a slower speed, which is characterized by a higher cadence and a longer swing phase during the gait cycle. These characteristics result in higher gyroscope drift; therefore, the IMU's accuracy in foot kinematics measurement might be specific to the walking abilities and characteristics of the participants analyzed. For this reason, for IMUs to be used as a clinical tool to assess gait for drop foot patients and evaluate the effects of FES, their accuracy needs to be investigated for this specific population.

The aim of this study is to evaluate the accuracy of IMU sensors to estimate foot angles in both the frontal and sagittal planes in order to establish their potential to analyze gait in healthy and impaired participants, specifically to help evaluate the effects of FES on drop foot patients in clinical settings. Based on the results from previous studies, we hypothesized that the accuracy of IMUs would allow the detection of foot kinematics and the improvements associated with the use of FES.

## 2. Methods

### 2.1. Participants

Thirteen unimpaired participants (seven women and nine men, age: 43.6 ± 10.8 years) and nine affected by drop foot (five women and four men, age: 58.4 ± 11.7 years) volunteered to take part in the study after ethical approval from Sheffield Hallam University Ethics Committee. The number of participants represents a convenient sample of patients and staff attending The National Clinical FES Centre at Salisbury District Hospital on the dates of data collection, with drop foot participants selected among patients suitable for the use of FES and under clinical evaluation at the Centre.

### 2.2. Experimental set-up

**Figure 1:**
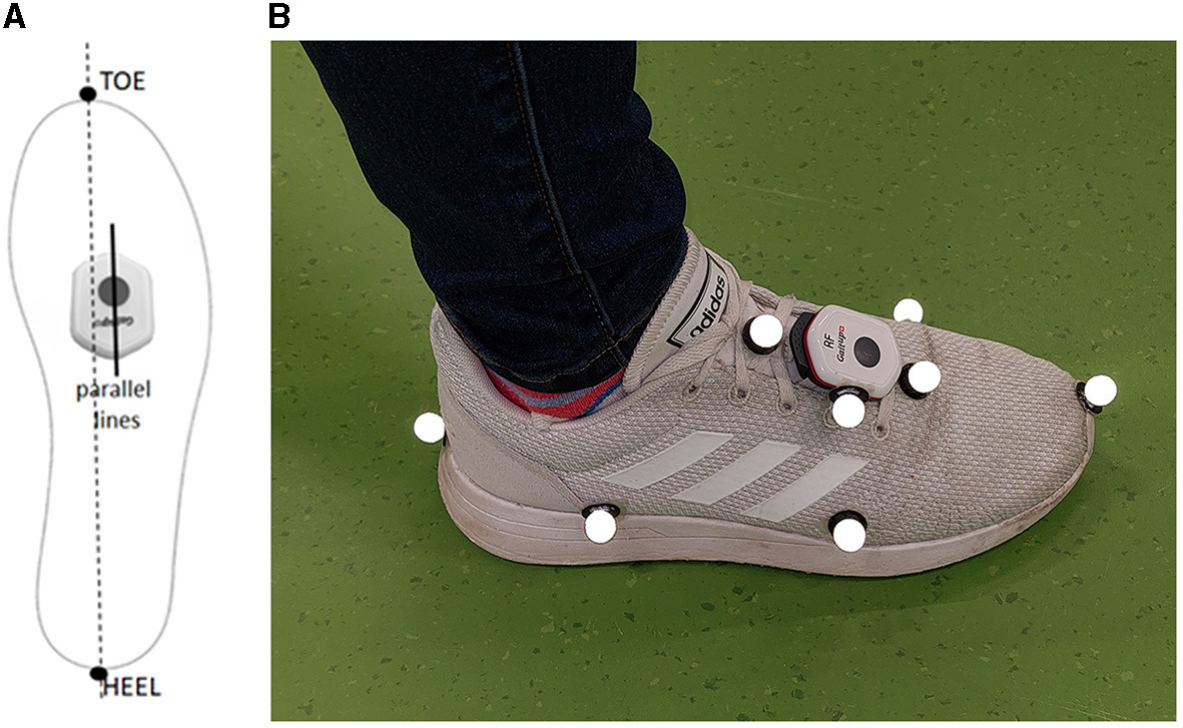
Sensor's alignment with the virtual heel-to-toe line of the foot **(A)**, and marker and Gait Up sensor locations on the right foot **(B)**.

Two Gait Up wireless sensors (Physilog 6, Gait Up SA, Lausanne, Switzerland) were used. The sensors were configured to set the accelerometer and gyroscope ranges at 16 g and 2,000°s^−1^, respectively, and to record at a sample frequency of 256 Hz. The sensors were attached to the participants' shoes over the laces through a clip provided with the sensors, aligning them with the virtual heel-to-toe line of the foot (Figure 1A). Gait Up IMUs have previously been validated to provide spatio-temporal parameters, foot clearance, and foot dorsi-plantar flexion angles on participants either healthy or affected by neurological conditions (Mariani et al., 2013; Brégou Bourgeois et al., 2014; Schwameder et al., 2015; Lefeber et al., 2019; Falbriard et al., 2020; Zhou et al., 2020); however, the sensors do not provide foot inversion-eversion angle.

An eight-camera portable optoelectronic motion capture system (Qualisys Miqus M3, Qualisys AB, Gothenburg, Sweden) sampling at 200 Hz was used to track the trajectories of sixteen markers, eight attached to each shoe of each participant (Figure 1B). Three markers on each shoe were aligned with the Gait Up sensor, and the remaining five markers were attached to anatomical landmarks (first and fifth metatarsal and heel) on the toe on the most forward point of the shoe and on the lateral side of the rear foot. The cameras were arranged to allow the tracking of markers over six meters of straight-line walking.

FES requires patients to wear footwear, as the switch that triggers stimulation is placed inside the shoe under the heel. Therefore, although the foot and shoe might move differently, the motion of interest in this study refers to the movement of the shoe, so sensors and markers were attached directly to the shoe.

### 2.3. Protocol

Unimpaired participants were asked to complete a total of six trials of straight-line walking, each with a different instruction. In the first three trials, participants were asked to walk at self-selected slow, normal, and fast speeds, where the normal walking speed was defined as each individual's own comfortable speed. Walking speeds were not controlled as they were grouped together in the analysis to provide a wider range of normal walking characteristics for healthy participants. For trials 4–6, participants were asked to walk simulating drop foot on the foot of their choice, first, without compensatory strategies and then with two different strategies that included exaggerating foot lift by increasing knee flexion and hip circumduction. The simulation of drop foot was used to analyze the agreement between the measurement systems for a wider range of walking characteristics compared to what would have been possible for the FES users alone. All healthy participants were experienced clinical professionals with a knowledge of drop foot.

Drop foot subjects were equipped with an FES system (ODFS Pace stimulator, Odstock Medical Ltd., Salisbury, UK) that had been set up by a specialist clinician and were asked to walk in a straight line with and without its assistance where possible. One of the participants was not able to walk without the use of FES and so performed one walk with stimulation from two channels and the second with stimulation from a single channel. Subjects walked with or without a walking aid according to how they felt more secure. All subjects were familiar with the use of FES.

A static trial (consisting of quiet standing with feet flat and in a natural stance facing in the direction of the walk) was recorded for each participant before the walking trials to zero the foot angles. A single trial was recorded for each condition during which 5–11 consecutive steps were captured. IMU sensors were switched off and on before each walking trial, with participants facing the direction of travel when the sensors were turned on.

### 2.4. Data processing

#### 2.4.1. Gait up

Gait Up raw data were read and synchronized through the provided MATLAB toolkit (Physilog 5 Research Toolkit). Raw gyroscope data were filtered with a first-order zero-lag low pass Butterworth filter with a cut-off frequency of 30 Hz and used to detect heel strike (HS), foot flat (FF), heel-off (HO), and toe-off (TO) according to Sabatini et al. (2005). In detail, an angular velocity threshold of 30°s^−1^ was set to detect the foot flat phase (FF to HO), and the angular velocity peaks were used to identify the beginning (TO) and end (HS) of the swing phase.

Foot angles were calculated from the unfiltered raw accelerometer and gyroscope. Raw data were measured in the sensor's local system, which was aligned relative to the foot. It was defined with the *x*-axis pointing toward the foot toe, the *z*-axis upwards, and the *y*-axis from right to left according to the right-hand rule. During the foot flat phase, the foot was assumed to be still on the ground, and, therefore, the acceleration measured by the sensor was considered close to gravity. This assumption was used to update the sensor orientation at each gait cycle and correct sensor drift. Sensor orientation was corrected only in the sagittal and frontal plane since azimuth corrections depend on magnetometer measurements, which are affected by the presence of ferromagnetic materials in an indoor clinical setting. In addition, the evaluation of foot kinematics in the transverse plane is of less importance in clinical measurements for drop foot patients. The sensor orientation relative to the laboratory coordinate system was defined by the rotation matrix *R*_0_ (Benoussaad et al., 2015):


$$R_{0}=\left[\begin{array}{ccc} 1 & 0 & 0\\ 0 & \text{cos}(\theta_{x}) & \text{sin}(\theta_{x})\\ 0 & -\text{sin}(\theta_{x}) & \text{cos}(\theta_{x}) \end{array}\right]*\left[\begin{array}{ccc} \text{cos}(\theta_{y}) & 0 & \text{sin}(\theta_{y})\\ 0 & 1 & 0\\ -\text{sin}(\theta_{y}) & 0 & \text{cos}(\theta_{y}) \end{array}\right]$$

where the angles θ_*x*_ and θ_*y*_ were calculated from the raw acceleration *a* = [*a*_*x*_ *a*_*y*_ *a*_*z*_] measured by the sensor during foot flat as:


$$\begin{array}{l} \theta_{x}=\text{atan}\left(\frac{a_{y}}{\sqrt{{a_{x}}^{2}+{a_{z}}^{2}}}\right)\\ \theta_{y}=\text{atan}\left(\frac{a_{x}}{a_{z}}\;\right). \end{array}$$

The sensor orientation during each gait cycle *n* was calculated using bidirectional strap-down integration through the following equation, starting from the rotation matrices *R*_0_ calculated at two successive foot flats:


$$\{\begin{array}{c} R_{gl}\left(t\right)=R_{gl}\left(t-\delta t\right)+R_{gl}\left(t-\delta t\right)*\Delta t*[w\left(t\right)\text{x}]\\ \forall \;t\;\in \;]FF_{n},\;midGC_{n}]\\ R_{gl}\left(t\right)=R_{gl}\left(t+\delta t\right)-R_{gl}\left(t+\delta t\right)*\Delta t*[w\left(t\right)\text{x}]\\ \forall \;t\;\in \;]midGC_{n},\;FF_{n+1}]\; \end{array}$$

where *R*_*gl*_(*t*) is the rotation matrix describing the orientation of the sensor in the global reference system at time *t*, Δt is the time interval between two successive samples, *midGC*_*n*_ is the sample representing the middle of the gait cycle *n*, equidistant to the two successive foot flat events *FF*_*n*_ and *FF*_*n*+1_, and [*w*(*t*)*x*] is the skew-symmetric matrix representing the cross-product operator of the angular velocity ω(t) measured by the gyroscope at time *t*.


$$\begin{array}{l} {}[w\;\text{x}]=\;\left[\begin{array}{ccc} 0 & -\omega_{z} & \omega_{y}\\ \omega_{z} & 0 & -\omega_{x}\\ -\omega_{y} & \omega_{x} & 0 \end{array}\right] \end{array}$$

Foot angles were calculated as Euler angles from the rotation matrix *R*_*gl*_ and the drift was corrected at each gait cycle *n* by balancing the forward and backward integration at the *midGC*_*n*_ sample.


$$\begin{array}{l} \theta_{midGC}=\frac{\theta \left(midGC_{n}\right)+\theta \left(midGC_{n}+1\right)}{2} \end{array}$$

$$\{\begin{array}{c} \theta \left(t\right)=\theta \left(t\right)+\frac{t-FF_{n}}{midGC_{n}-FF_{n}}*(\theta_{midGC}-\theta (midGC_{n}))\\ \forall \;t\;\in \;]FF_{n},\;midGC_{n}]\\ \theta \left(t\right)=\theta \left(t\right)+\frac{t-FF_{n+1}}{FF_{n+1}-midGC_{n}}*\left(\theta \left(midGC_{n}+1\right)-\theta_{midGC}\right)\\ \forall \;t\;\in \;]midGC_{n},\;FF_{n+1}] \end{array}$$

Foot angles were calculated relative to the laboratory coordinate system (i.e., relative to the ground) rather than the lower limb as a more important clinical measurement to evaluate the risk of falls and injuries associated with drop foot and the effect of FES.

#### 2.4.2. Motion capture

Marker trajectories were filtered with a first-order zero-lag low-pass Butterworth filter with a cut-off frequency of 30 Hz. Foot angles were calculated from the markers attached to anatomical landmarks instead of those aligned with the sensors to compare true foot movement instead of only the measurement systems. The angles were resampled to 256 Hz before being synchronized with those estimated from the Gait Up sensors using cross-correlation. The sequences of the entire foot angle obtained from the motion capture and the IMUs in the sagittal plane were cross-correlated using the *xcorr* function in MATLAB to measure the similarity between the two signals and determine the delay between them. Cross-correlation was used because the walking starting point was outside the motion capture volume. The alignment between the data from the two systems was visualized to confirm the synchronization.

### 2.5. Statistical analysis

The instantaneous synchronized data were compared using the root mean square error (RMSE) curve (mean of RMSE calculated at each time point). Bland Altman limits of agreement with 95% confidence intervals were used to compare the systems for maximum and minimum values, range of motion, and angle at HS as calculated at each gait cycle for both dorsi-plantar flexion and inversion-eversion. All data were compared overall and by dividing the trials into three groups: unimpaired participants walking at different speeds, unimpaired participants walking simulating drop foot with or without compensatory strategies, and drop foot patients walking with or without electrical stimulation. Bland Altman limits of agreement were also used to evaluate the accuracy of the IMUs in comparison to the gold standard system in determining foot angle changes in the sagittal and frontal planes following FES intervention. The dorsi-plantar flexion and inversion-eversion differences between drop foot patients walking with and without FES were calculated for each system and used for the comparison. All data were calculated and processed using MATLAB R2021b (Mathworks, Natick, MA, USA).

## 3. Results

In Table 1, the curve RMSE was reported to compare the instantaneous foot angles calculated in the sagittal and frontal planes of motion from the Gait Up sensors data with those obtained through the motion capture. The results were reported for four different comparisons obtained considering separately the trials for unimpaired participants walking at different speeds (*n* = 39), for unimpaired participants walking simulating drop foot (*n* = 39), and for FES patients (*n* = 18) and considering the trials from all the participants (*n* = 96). For each participant group, higher accuracy was obtained in the estimation of dorsi-plantar flexion angles compared to inversion-eversion. Among the groups, the estimation of dorsi-plantar flexion and inversion-eversion was higher for the FES patients group.

**Table 1:** RMSE curve.

	**Dorsi-plantar flexion (°)**	**Inversion-eversion (°)**
Unimpaired participants' normal walk	4.88	5.94
Unimpaired participants' simulated drop foot	4.73	6.72
FES patients	3.68	4.55
Overall	4.58	5.97

The RMSE between motion capture and IMUs was calculated at each time point, and the mean was calculated. These values are a measure of the accuracy of IMUs to estimate instantaneous foot angles in comparison to the gold standard measurement method.

In Tables 2, 3, Bland–Altman limits of agreement are reported for dorsi-plantar flexion and inversion-eversion foot angles, respectively. The comparison was performed for maximum, minimum, and range of motion values calculated from each complete gait cycle (HS to HS) and for the angle at each detected heel strike. Data were evaluated overall (*n* = 612) and separately for unimpaired participants walking at different speeds (*n* = 196), unimpaired participants walking simulating drop foot (*n* = 261), and FES patients (*n* = 155).

**Table 2:** Bland–Altman limits of agreement (LOA) comparison for dorsi-plantar flexion foot angle.

		**Mean (°)**	**SD (°)**	**Upper LOA (°)**	**Lower LOA (°)**
Unimpaired participants' normal walk	Max	4.36	4.12	12.43	−3.72
	Min	−4.71	3.72	2.58	−11.99
	Rom	9.06	3.19	15.32	2.81
	At HS	−0.94	4.29	7.48	−9.35
Unimpaired participants' simulated drop foot	Max	4.80	4.91	14.43	−4.83
	Min	−2.91	5.25	7.39	−13.21
	Rom	7.71	4.68	16.88	−1.46
	At HS	−0.96	4.51	7.89	−9.81
FES patients	Max	3.56	3.54	10.49	−3.38
	Min	−3.39	3.66	3.79	−10.56
	Rom	6.95	3.23	13.27	0.63
	At HS	−1.66	3.40	5.00	−8.32
Overall	Max	4.35	4.37	12.91	−4.22
	Min	−3.61	4.49	5.19	−12.40
	Rom	7.95	3.98	15.76	0.14
	At HS	−1.12	4.20	7.11	−9.35

The systems were compared for maximum (max) and minimum (min) values, range of motion (rom), and the angle at heel strike (HS). The mean and standard deviation (SD) were calculated from the differences between motion capture and IMU measurements. The 95% limits of agreement were calculated as mean ± 1.96 SD to determine the upper and lower LOA within which 95% of the differences between the systems were expected to fall.

**Table 3:** Bland–Altman limits of agreement (LOA) comparison for inversion-eversion foot angle.

		**Mean (°)**	**SD (°)**	**Upper LOA (°)**	**Lower LOA (°)**
Unimpaired participants' normal walk	Max	−2.72	7.50	11.98	−17.41
	Min	1.36	3.82	8.85	−6.12
	Rom	−4.08	6.77	9.19	−17.35
	At HS	1.19	4.46	9.94	−7.56
Unimpaired participants' simulated drop foot	Max	−0.15	7.83	15.20	−15.51
	Min	1.62	6.08	13.53	−10.29
	Rom	−1.77	6.69	11.34	−14.89
	At HS	2.41	5.93	14.02	−9.21
FES patients	Max	0.46	6.15	12.51	−11.59
	Min	1.36	3.78	8.76	−6.05
	Rom	−0.89	5.47	9.84	−11.62
	At HS	−0.14	3.56	6.84	−7.11
Overall	Max	−0.82	7.44	13.76	−15.40
	Min	1.47	4.90	11.07	−8.13
	Rom	−2.29	6.55	10.54	−15.12
	At HS	1.38	5.05	11.28	−8.52

The systems were compared for maximum (max) and minimum (min) values, range of motion (rom), and the angle at heel strike (HS). The mean and standard deviation (SD) were calculated from the differences between motion capture and IMU measurements. The 95% limits of agreement were calculated as mean ± 1.96 SD to determine the upper and lower LOA within which 95% of the differences between the systems were expected to fall.

The agreement between the motion capture system and IMUs in detecting changes in foot angles following FES intervention was evaluated with Bland–Altman limits of agreement. The results are reported in Table 4.

**Table 4:** Bland–Altman limits of agreement (LOA) to evaluate the accuracy of the IMUs in comparison to the gold standard system in determining foot angle changes in the sagittal and frontal planes following FES intervention.

		**Mean (°)**	**SD (°)**	**Upper LOA (°)**	**Lower LOA (°)**
Dorsi-plantar flexion	Max	−0.41	3.07	5.61	−6.44
	Min	−1.28	3.21	5.01	−7.57
	Rom	0.86	3.01	6.76	−5.04
	At HS	−1.18	2.22	3.16	−5.53
Inversion-eversion	Max	0.48	7.85	15.86	−14.89
	Min	1.55	4.22	9.82	−6.73
	Rom	−1.06	4.85	8.44	−10.56
	At HS	2.47	4.86	11.98	−7.05

The dorsi-plantar flexion and inversion-eversion differences between drop foot patients walking with and without FES were calculated for each system and used for the comparison of maximum (max) and minimum (min) values, range of motion (rom), and the angle at heel strike (HS). The mean and standard deviation (SD) were calculated from the differences between motion capture and IMU measurements. The 95% limits of agreement were calculated as mean ± 1.96 SD to determine the upper and lower LOA within which 95% of the differences between the systems were expected to fall.

## 4. Discussion

The current study aimed to evaluate the potential of IMU sensors to be used as clinical tools to analyze gait in healthy and impaired subjects and to help understand the effect of FES on drop foot patients in clinical settings. Dorsi-plantar flexion and inversion-eversion angles were estimated from the raw data of two Gait Up wireless sensors, one on each foot, and compared with the measurements from a gold standard measurement system. The importance of an accurate estimation of foot angles in the sagittal and frontal planes for drop foot patients is justified by the need to understand patients' responses to treatment or rehabilitation. The aim of understanding these responses is related to reducing the risk of tripping, falling, and ankle injury due to the limited ability in foot dorsiflexion and the poor placement of the foot on the ground at initial contact. In addition, an accurate estimation of foot angles helps to evaluate the effect of FES and electrode placement needed to achieve a well-balanced dorsiflexion without excessive inversion or eversion.

The results in Table 1 show lower errors when predicting dorsi-plantar flexion angles in comparison to inversion-eversion for each subject group, with the difference being even greater when considering that the total range of motion is between 65° and 75° in the sagittal plane and ~35° in the frontal plane (Grimston et al., 1993). Inversion-eversion is a complex movement involving multiple bones and joints; therefore, the motion tracked by a single sensor placed on the midfoot represents a simplification with consequent higher errors in the prediction of instantaneous values. Among the subject groups considered, Table 1 shows lower errors for dorsi-plantar flexion and inversion-eversion in the FES patients' group. This might be explained by small inaccuracies found in the detection of gait events for unimpaired participants walking at higher speeds and simulating drop foot when using the algorithm proposed by Sabatini et al. (2005), which was based on angular velocity data from participants walking at a normal pace. These inaccuracies affect drift correction and might explain the smaller errors in the range of motion of dorsi-plantar flexion and inversion-eversion angles in FES patients compared to the other two groups (Tables 2, 3).

Drop foot patients have limited ability to dorsi-plantar flex the foot; therefore, the evaluation of the range of motion in the sagittal plane represents an important clinical measurement. Overall, errors of 7.95° ± 3.98° were obtained in this measurement, which was mainly related to an underestimation of the angle peaks. The errors obtained in this study are higher compared to previous studies from Kanzler et al. (2015) and Seel et al. (2015). Kanzler et al. estimated a dorsi-plantar flexion continuous angle with a mean difference of 2.49 ± 1.21° for healthy participants aged between 16 and 80 walking at three self-selected speeds. Seel et al. compared the angles in the sagittal plane for two healthy participants walking barefoot at slow and fast paces and two transfemoral amputees walking with a leg prosthesis at self-selected speed, and the authors reported the mean difference for each subject, wherein 3.79 ± 0.73° was the maximum error obtained. In these studies, for healthy subjects, the sensors were placed either on the lateral side of participants' shoes underneath the lateral malleolus or on the instep of the bare feet. The higher errors found in our study might be related to the sensor mounting site, which was over the laces rather than on the lateral aspect of the shoe. This might have caused more sensor movement relative to the foot or shoe. This attachment location was chosen based on the most recent recommendation from Gait Up. However, future studies should compare the two different locations when evaluating foot kinematics in the sagittal and frontal planes.

The measurement of foot angles at HS is useful for understanding initial foot placement on the ground, and it can inform clinicians about the effect of rehabilitation or treatment procedures. IMU's accuracy in the evaluation of foot angles at HS was assessed, and the errors in comparison to the gold standard system are reported in Tables 2, 3. Overall, errors of −1.12° ± 4.20° and 1.38° ± 5.05° were obtained for dorsi-plantar flexion and inversion-eversion, respectively. Previous research evaluated the dorsi-plantar flexion angle at foot strike for healthy children and those with cerebral palsy (Brégou Bourgeois et al., 2014), reporting errors of 0.5° ± 2.9°; for healthy adults walking normally or with an induced limping condition at self-selected slow, neutral, and fast speeds (Schwameder et al., 2015), reporting errors ranging between 2.0° ± 1.2° and 8.3° ± 4.2° between the different conditions; and, for post-stroke subjects walking with FES, reporting mean errors of 3.39° (Sijobert et al., 2018).

The IMU accuracy in determining foot angle improvements associated with the use of FES on drop foot patients was evaluated against the gold standard system. The dorsi-plantar flexion and inversion-eversion differences between drop foot patients walking with and without FES were calculated for each system and used for the Bland–Altman comparison. The results are reported in Table 4. In the literature, significant improvements associated with the use of FES were reported when analyzing the difference in peak dorsiflexion during swing and ankle dorsiflexion at HS with a motion capture system (Scott et al., 2013; Van der Linden et al., 2014; Pool et al., 2015). In this study, peak dorsiflexion during swing was reported as the minimum value calculated during dorsi-plantar flexion, and the limits of agreement obtained (5.01° and −7.57° for upper and lower limit, respectively) were too wide to be able to detect improvements ranging between 2.6° and 8.1° found in previous studies (Scott et al., 2013; Van der Linden et al., 2014; Pool et al., 2015). Similarly, increases in ankle dorsiflexion at initial contact were found to range between 3.3° and 11.9° (Scott et al., 2013; Van der Linden et al., 2014; Pool et al., 2015), and the limits of agreement found in this study were not good enough (3.16° and −5.53° for upper and lower limits, respectively) to detect these changes. No studies were found in the literature reporting improvements in inversion-eversion following FES intervention; however, from the values reported in Table 4, it is suggested that IMUs would not be able to accurately detect changes in foot angle in the frontal plane.

Limitations of this study are associated with sensor attachment. IMUs were attached to the participants' shoes through a clip provided to place the sensor on the laces. However, due to the different types of shoes worn, the use of the clip was not always possible, and in some cases, the sensor was attached directly to the shoe with tape. This might have affected the movement of the sensors compared to the surrounding markers, especially in the frontal plane. In addition, the algorithm used to detect gait events needs improvements when fast walking is analyzed.

Despite inversion-eversion foot angles previously being estimated using IMUs, to the best of our knowledge, this is the first study comparing this measurement with a gold standard method. The results show that IMUs are currently not able to accurately assess foot movements in the frontal plane during gait for a range of walking abilities. In addition, the evaluation of changes in foot angles following FES intervention showed low agreement between IMUs and motion capture in the sagittal plane. Therefore, our initial hypothesis that IMUs were accurate enough to detect foot kinematics and the improvements associated with the use of FES is rejected. More work is necessary before IMUs can be used in clinical settings to evaluate foot kinematics for drop foot patients.

## 5. Conclusion

This study aimed to evaluate the accuracy of IMU sensors in comparison to other motion capture systems to estimate foot kinematics in the frontal and sagittal planes and to determine if IMUs are suitable for clinical use to detect the improvements associated with the use of FES in drop foot patients. The results of this study showed low agreement between the systems, in particular for the evaluation of inversion-eversion and changes in foot angles following FES intervention. Therefore, it can be concluded that IMUs are currently not an alternative to motion capture systems to analyze gait in drop foot patients in clinical settings.

## Data availability statement

The raw data supporting the conclusions of this article will be made available by the authors, without undue reservation.

## Ethics statement

The studies involving humans were approved by Sheffield Hallam University Ethics Committee. The studies were conducted in accordance with the local legislation and institutional requirements. The participants provided their written informed consent to participate in this study.

## Author contributions

Fd'A performed the data analysis and wrote the first draft of the manuscript. Fd'A and BH interpreted the results. BH contributed to the manuscript revision. All authors contributed to the conception and design of the study and collection of data. All authors read and approved the submitted version.
